# Influence of *DUX4* Expression in Facioscapulohumeral Muscular Dystrophy and Possible Treatments

**DOI:** 10.3390/ijms24119503

**Published:** 2023-05-30

**Authors:** Elisa Duranti, Chiara Villa

**Affiliations:** School of Medicine and Surgery, University of Milano-Bicocca, 20900 Monza, Italy; e.duranti@campus.unimib.it

**Keywords:** facioscapulohumeral muscular dystrophy, DUX4, muscle differentiation

## Abstract

Facioscapulohumeral muscular dystrophy (FSHD) represents the third most common form of muscular dystrophy and is characterized by muscle weakness and atrophy. FSHD is caused by the altered expression of the transcription factor double homeobox 4 (DUX4), which is involved in several significantly altered pathways required for myogenesis and muscle regeneration. While *DUX4* is normally silenced in the majority of somatic tissues in healthy individuals, its epigenetic de-repression has been linked to FSHD, resulting in *DUX4* aberrant expression and cytotoxicity in skeletal muscle cells. Understanding how *DUX4* is regulated and functions could provide useful information not only to further understand FSHD pathogenesis, but also to develop therapeutic approaches for this disorder. Therefore, this review discusses the role of DUX4 in FSHD by examining the possible molecular mechanisms underlying the disease as well as novel pharmacological strategies targeting *DUX4* aberrant expression.

## 1. Introduction

**Figure 1:**
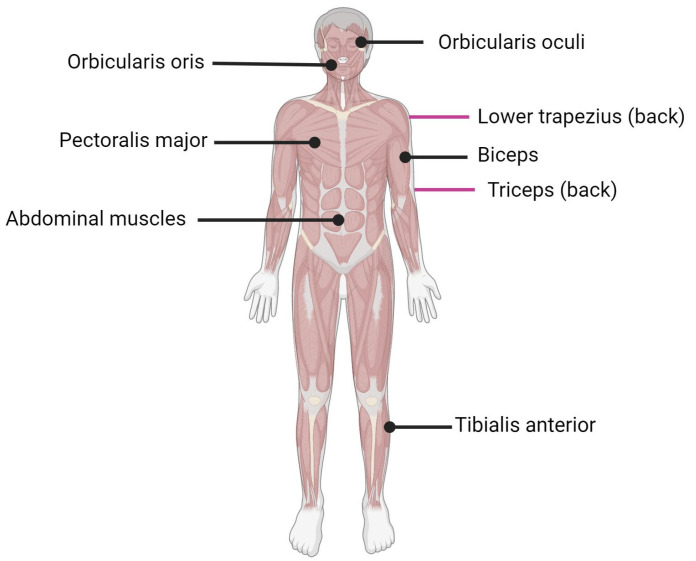
Muscles affected by FSHD. Black lines indicate muscle located in front of the body; purple lines indicate muscle located on the back of the body.

Facioscapulohumeral muscular dystrophy (FSHD) is the third most common form of muscular dystrophy after myotomic and Duchenne muscular dystrophy with an estimated incidence of 1:15,000 to 1:20,000 [1]. FSHD affects the facial (facio) and shoulder girdle (scapulo-humeral) muscles, hence the name facioscapulohumeral dystrophy (Figure 1). The disease usually progresses from the upper to lower extremities, with the subsequent involvement of the anterior distal muscles of the leg. Muscle malfunction can impair high-frequency hearing and retinal telangiectasias [2]. Both sexes of all ages can be affected by FSHD [3,4], although men may develop an earlier onset than women in the case of mosaicism [5,6]. The symptoms typically appear during adolescence, but the first onset and severity might vary greatly, and in the most severe cases, the symptoms may appear in infancy [7]. Currently, there are no treatments for FSHD, and available therapeutic options include improvements in daily functioning, surveillance for extramuscular complications, and minimizing discomfort and tiredness [8].

After intensive research, there is a consensus that FSHD is caused by the aberrant expression of the full-length isoform of double homeobox transcription factor (DUX4), particularly in skeletal muscle nuclei [9]. *DUX4* is normally expressed in the early stages of development in stem cells and germ lines, especially in the testis, while it is repressed via a repetition-mediated epigenetic silencing (methylation) mechanism [10] during cell differentiation [11] and in most adult somatic tissues, including muscle [12,13], except for the thymus [14] and keratinocytes [15]. However, the precise mechanism by which this gene induces dystrophic changes, as well as the changes themselves induced at the cellular level, are still controversial and under investigation [12,15]. Tassin et al., have proposed a dynamic model for DUX4 protein expression in FSHD myotubes. In this model, the DUX4 transcription factor, initially expressed in few nuclei, diffuses in many nuclei of the myotube and thus activates a transcriptional deregulation cascade in each nucleus to which it has diffused. The presence of a dystrophic phenotype causes compromised muscle tissue. The researchers hypothesized that even low levels of DUX4 could result in the formation of amorphous muscle cells [16]. The relatively higher presence of DUX4 in myotubes as opposed to proliferating myoblasts may imply that *DUX4* transcription is induced during differentiation.

This review summarizes pathological muscle differentiation focusing on the role played by DUX4 in FSHD muscle in order to better understand the pathophysiology of the disease and to provide novel possible therapeutic strategies targeting *DUX4* aberrant expression.

## 2. Myogenesis and Muscle Regeneration

Skeletal muscle, one of the three major muscle types, is a contractile tissue responsible for movement, maintaining posture, supporting soft tissues, and maintaining temperature. Tendons are bundles of collagen fibers that connect skeletal muscles to bones, skin, and other muscles. Skeletal muscle is composed of multinuclear cells called myofibers [17], which are formed by the fusion of myoblasts during development [18]. When a muscle is injured, it activates a complex response that leads to tissue regeneration [19,20,21]. Skeletal muscle regeneration is primarily mediated by satellite cells (SCs) that receive signals from the surrounding environment [17,22,23,24], which replenish myogenic progenitor cells and differentiate into new myofiber for muscle repair in response to injury [17,25,26]. Muscle regeneration and differentiation are initiated with the modulation of the expression of certain genes and proteins: myogenic regulatory factors (MRFs, summarized in Table 1).

**Figure 2:**
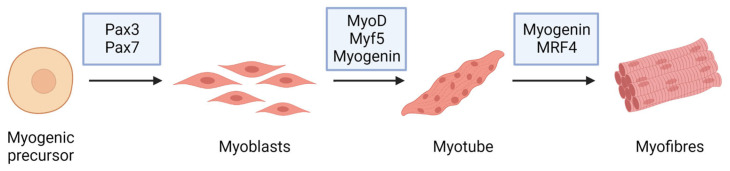
Myogenic markers and stage-specific expression of the major proteins involved in muscle differentiation.

The term myogenesis refers to the complex cellular process that mediates the formation of a skeletal muscle fiber starting from a myogenic precursor during embryonic development, as in adult tissue repair [29] (Figure 2). The myotomy level, which is the sub-medial area of the somite, formed by cells that, splitting from it, stretch and gradually differentiate into myoblasts, is where myogenesis starts in the first week of embryonic development [30]. Their fusion in myotubes occurs before the development of mature muscle fibers so that the entire locomotor apparatus originates from the myotomes of the various subtypes. Myoblasts and muscle fibers in the maturation phase are also surrounded by a fibroblastic connective tissue scaffold that guides the development and spatial organization [31]. Other factors, such as the surrounding environment and paracrine factors, actively control and regulate the process, activating muscle-specific genes. The coding genes for the factors responsible for myogenic differentiation begin to express themselves in a coordinated manner in proliferating myoblasts [32]. MRFs are products of these genes (Figure 2): their expression is mediated by paracrine factors and the factors present in the surrounding microenvironment, which first activate the paired box gene 3 (Pax3) transcriptional factor [33].

Among the major MRFs are myoblast determination protein 1 (MyoD) and myogenic factor 5 (Myf5) transcription factor proteins belonging to the family of basic helix–loop–helix (bHLH) myogenic proteins, which are responsible for myoblastic commitment and myogenic regulatory factor 4 (MRF4, also known as Myf6) and myogenin, which are required to keep myotubes differentiated [28]. Pax3 directly activates the transcription of MyoD and Myf5. Myoblasts are myotome cells that produce bHLH proteins and can proliferate in the presence of specific growth factors. The depletion of these factors is responsible for cell proliferative arrest, fibronectin secretion, and the expression of integrin receptors. The fibronectin–integrin adhesion signal is required for myoblasts to start the differentiation process. Cell–cell recognition is the event that causes the arrest of the cell cycle [34,35].

This may thus begin cell fusion, giving rise to myotubes. Cells at this point are unable to respond to mitogenic stimuli, and during the late stages of the process, myoblasts that have already fused secrete factors, promoting the fusion of additional myoblasts to the myotube in formation. During embryonic myogenesis, mesoderm-derived structures generate the first muscle fibers of the body proper, and in subsequent waves, additional fibers are generated along these template fibers [36,37]. Although the mesoderm is the only germ layer of a trilaminar embryo capable of generating skeletal muscle, the exact sites of origin and regulators of body muscle vary, depending on the group of specific embryonic muscle, i.e., dorsal or ventral trunk muscle, limb muscle, and head or neck muscle [38]. Pax3-positive myogenic stem cells, named SCs, are located between the basal lamina and sarcolemma of associated myofibers which ensure adult muscle growth. These cells can both replicate themselves (self-renewal) and, after activation, escape from a quiescent state and give rise to proliferating myoblasts by re-entering the cell cycle.

Skeletal muscle can regenerate itself on a daily basis as well as in response to injury [39]. This ability is due, at least partly, to the adult stem cell population that has been named SCs because of their location at the periphery of mature skeletal myofibers. Muscle regeneration depends on a balance between pro-inflammatory and anti-inflammatory factors, which determine whether the damage is repaired with muscle fiber replacement and the reconstitution of a functional contractile apparatus, or with scar formation [40]. Muscle tissue repair following damage can be thought of as a two-step process with two interdependent phases: degeneration and regeneration. 

The first event, degeneration, is characterized by the disruption of myofibers. In the early stages of muscle injury, inflammatory cells usually infiltrate the damaged muscle. Among the primary immune cells involved, macrophages play a critical role: after the infiltration, macrophages phagocytose cellular debris and remove disrupted myofilaments, other cytosolic structures, and the damaged sarcolemma. Following injury, muscle repair processes are activated and quiescent SCs enter a massive proliferation phase, allowing the myogenic cell population to expand. This proliferation is characterized by an asymmetric cell division in which SCs replicate themselves for their self-renewal and can generate proliferating myoblasts for the development of the new muscle fibers. Myoblasts can differentiate and fuse in order to repair or to generate new fibers [41]. Muscle regeneration is regulated by a family of muscle-specific, basic helix–loop–helix transcription factors called MRFs, including MRF4, myogenin, MyoD, and Myf5. After muscle injury, Myf5 and MyoD are typically the first MRFs to be expressed in the regenerating muscle cells, followed by myogenin, and finally MRF4. However, MyoD and Myf5 play different roles in the process of muscle regeneration. While MyoD promotes SCs’ progression to terminal differentiation, Myf5 promotes SCs’ self-renewal [28].

## 3. Myoblast Fusion

Myoblast fusion is the process that results in the generation of syncytial muscle cells. It can occur between myoblasts (primary fusion) and myotubes (secondary fusion), and it can also happen during muscle regeneration. Injury is sufficient to activate SCs, which can produce new myoblasts after an asymmetric cellular division, necessary to maintain the SC pool [42]. Mechanistic studies of these components suggest that muscle cells go through at least three consecutive steps before forming a fusion pore [43]. Muscle cell fusion begins when myoblasts exit the cell cycle. Myoblasts will proliferate without differentiating if growth factors (particularly fibroblast growth factors) are present. The second step is cell recognition, which involves aligning the myoblasts into chains. The third step is the cell fusion event itself. Recent studies in a variety of model organisms have uncovered many molecular components required for myoblast fusion. Many steps in this process are facilitated by the actin cytoskeleton, for example. Myoblasts need cytoskeletal shape changes to migrate toward their sites of fusion, and a reorganization of the actin cytoskeleton is required for the following steps: fusion recognition, adhesion, and vesicle transport [44]. Furthermore, although glycolipids and cholesterol are less abundant, they play an important role in regulating membrane polarity and fluidity [45]. Moreover, cholesterol is required for the formation of specialized membrane regions responsible for the regulation of fusion signaling, such as lipid rafts and caveolae [46]. Another fundamental molecule class is represented by the proteins involved in recognition and adhesion. This step necessitates the use of specific integrin family members and cell adhesion molecules (CAMs). The recognition is also mediated by cell membrane glycoproteins, including several cadherins [47]. It plays an important role in mammalian myoblast regeneration, but it has also been found in developing muscle, even if its pathway expression is more visible after the initial fusion steps have been completed. Moreover, M-cadherin (M-cad) is also expressed in SCs and the sarcolemma. Once fusion occurs, M-cad signaling is switched off by M-cad movement into caveolae. M-cad is thus sequestered from the plasma membrane and subsequently transported to the proteasome for degradation [48]. Furthermore, recent studies have identified specific cell signaling pathways whose activation results in the expression of genes required for the fusion process and cytoskeleton rearrangement regulation.

## 4. Genetics of FSHD

On the basis of their underlying epigenetic mechanism, two well-defined subtypes of FSHD exist, namely FSHD1 and FSHD2. Both of them often show an autosomal dominant pattern of inheritance and result in chromatin relaxation and abnormal *DUX4* expression in skeletal muscle, leading to progressive muscle weakness and atrophy [49,50].

**Figure 3:**
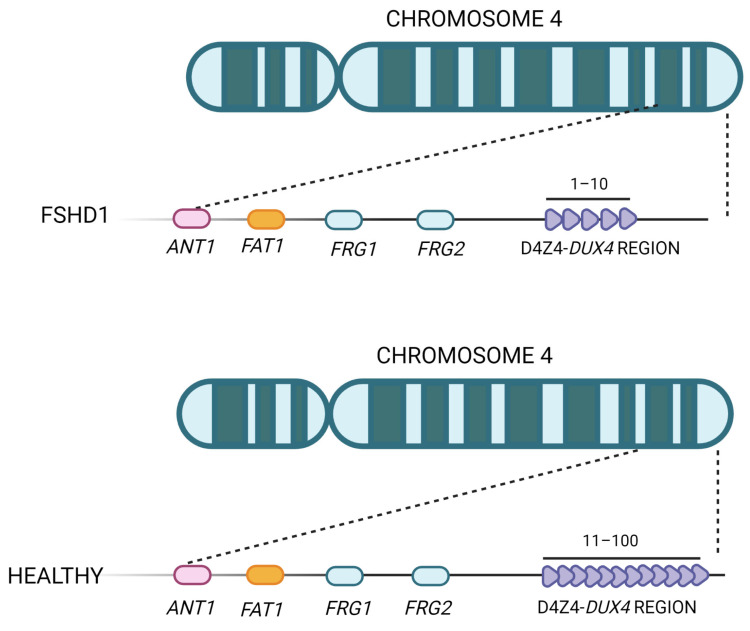
Representation of the FSHD locus. The D4Z4 repeat array is located in the subtelomere of chromosome 4 and can vary between 11 and 100 copies in healthy individuals. In FSHD patients, the structure of D4Z4 adopts a more open configuration and has fewer copies (between 1 and 10).

FSHD1 represents the most common form, accounting for about 95% of all FSHD cases, and is caused by the partial deletion (shortening or contraction) of the macrosatellite D4Z4 repeat, located in the subtelomeric region of chromosome 4 (4q35) (Figure 3) [16,51,52,53]. The D4Z4 macrosatellite, consisting of repeated units of 3.3 kb, is highly polymorphic: in the healthy population, the number of copies varies between 11 and 150, whereas in affected individuals, the number of copies ranges between 1 and 10. This contraction results in a partial loss of D4Z4 DNA methylation, which ultimately leads to *DUX4* transcription in skeletal muscle [54]. The severity of the disease increases as the number of repetitions decreases [55,56]. The decrease in D4Z4 units leads to chromosome relaxation and hypomethylation, allowing DUX4 transcription in muscle cells [53]. In addition to *DUX4*, additional genes located in the 4q35 region proximal to the D4Z4 repeat array, such as the FSHD region genes 1 and 2 (*FRG1*, *FRG2*), adenine nucleotide translocator 1 (*ANT1*) and FAT atypical cadherin 1 (*FAT1*), seem to be inappropriately overexpressed in affected muscles [57], but their role in both the onset and severity of disease is still controversial. *FRG1* has been considered a candidate gene because of its development of a phenotype similar to that of FSHD in a murine model overexpressing *FRG1* [57], and it has been linked to muscle development [10]. Indeed, while *FRG1* is subject to extreme variability, it has been found to be upregulated in affected patients. *FRG2* is 37kb proximal to D4Z4 and is specifically upregulated in FSHD muscle cells that are differentiating [58]. This gene does not appear to be expressed in some FSHD patients who have an extended deletion at the proximal portion of the macrosatellite, implying that its dysregulation is more likely the result of epigenetic changes than a direct cause of pathology [59,60]. In support of this, *FRG2* overexpression in mouse models did not result in the development of muscular dystrophy [61,62]. *ANT1* encodes a mitochondrial homodimeric protein that is localized asymmetrically on the inner mitochondrial membrane. The dimer forms a membrane channel through which ADP can pass from the matrix to the cytoplasm, being thus essential for cellular oxidative metabolism. ANT1 protein levels appear to be higher in FSHD muscles than in healthy controls or patients with Duchenne muscular dystrophy [57], making muscle cells more susceptible to oxidative stress and apoptosis [63]. While the involvement of *FRG1*, *FRG2*, and *ANT1* in FSHD pathogenesis is still debated, the role of the *FAT1* gene in this disease has been confirmed by independent studies [64,65,66]. FAT1 is a member of the cadherin-like protein family and is involved in the regulation of tissue growth, morphogenesis, and polarity during development [67]. The first association between *FAT1* and FSHD was reported in *Fat1*-deficient mice, which showed muscular and non-muscular phenotypes resembling FSHD symptoms and pathological features [64]. Other authors observed a lower expression of *FAT1* in diseased adult muscles than in matched controls, which did not appear to be regulated by DUX4 [65]. They also found that *FAT1* is expressed at lower levels in early-stage FSHD-affected muscles compared to later-stage or unaffected muscles in control fetal human biopsies or developing mice embryos [65]. Additional experimental research and case reports have further confirmed *FAT1* as a gene involved in disease onset and severity [64,66]. However, further in-depth studies are needed to clearly understand its role along with the cellular and molecular mechanisms leading to its altered expression in FSHD cells [64,66,68].

The rarer FSHD2 form, which accounts for the remaining 5% of FSHD patients, has been attributed to variants in D4Z4 chromatin repressors, mainly occurring within the structural maintenance of the chromosomes flexible hinge domain-containing 1 (*SMCHD1*) gene encoding a chromatin remodeling factor important for DNA methylation [69,70]. Interestingly, *SMCHD1* mutations have been also reported to act as modifiers of disease severity in patients with FSHD1 [71,72], suggesting that FSHD type 1 and 2 form a disease continuum instead of separate entities [73]. Rare heterozygous variants in the DNA methyltransferase 3β (*DNMT3B*) gene have been also associated with FSHD2 manifestation and penetrance [74]. Intriguingly, biallelic *DNMT3B* mutations are also responsible for immunodeficiency, centromeric instability, and facial anomalies syndrome type 1 (ICF1) [75]. Moreover, a homozygous mutation in the *LRIF1* gene (encoding a ligand-dependent nuclear receptor-interacting factor 1) that suppresses the long isoform of this protein has been recently detected in a patient with FSHD2.

Rare FSHD cases have been linked to uncommon DNA changes leading to D4Z4 chromatin relaxation, thus allowing for *DUX4* transcription [76,77,78]. Intronic mutations in *SMCHD1* influencing mRNA splicing [79], partial deletions of the D4Z4 macrosatellite repeat array extending proximally into surrounding non-D4Z4 sequences [61,80], and small duplications of the D4Z4 macrosatellite repeat region [78,81] are examples of these alterations.

However, disease-causing mutations have not been found in a small subset of FSHD patients with a normal-sized but transcription-permissive D4Z4 macrosatellite repeat, suggesting the existence of additional FSHD-related modifiers, probably other D4Z4 chromatin modifiers [82].

FSHD has been proven to be a genetically heterogeneous disorder involving both genetic and epigenetic alterations. A thorough genomic analysis of the 4q35 region resulted in the identification of several haplotypes. In particular, 15 single-nucleotide polymorphisms (SNPs) were discovered in a region near D4Z4 (the D4F10S1 region), and a second large region with sequence variants (alleles A, B, and C) was also identified distal to D4Z4. Based on these differences, 4q alleles can be classified into 18 haplotype variants, with macrosatellite deletions being pathogenic in only a few of them (4qA161, 4qA159, and 4qA168) [55,83]. In permissive haplotypes (the 4qA allele), SNP causes the appearance of an ATTAAA polyadenylation signal. In non-permissive haplotypes, the sequence is instead ATCAAA, which is a non-functional polyadenylation signal. Transfection experiments that introduced the functional site into non-permissive alleles and removed it from permissive alleles confirmed the importance of the polyadenylation site [83]. The presence of a stable *DUX4* transcript was detected solely in the presence of the polyadenylation site and appears to directly affect the etiopathogenesis of FSHD. D4Z4 deletions in permissive alleles, on the other hand, are insufficient to cause FSHD; in fact, asymptomatic carriers have been observed. This suggests that these haplotypes are just a permissive condition for dystrophy development. As a result, the great number of D4Z4-homologous sequences found in the genome, along with the complexity of the subtelomeric 4q region, have always made the understanding of the molecular mechanisms underlying FSHD particularly difficult [55,84].

## 5. DUX4

DUX4 is formed by the two neighboring homeodomains of 60 amino acids, HD1 and HD2, which are located in the N-terminal part and are responsible for the binding of sequence-specific DNA. DUX4 has three nuclear localization signals (NLS) located in the homeodomain region and at the C-terminal [85]. The binding of DUX4 to DNA is critical for its physiological and pathological functions. The significance of DUX4–DNA binding is being studied because it could lead to the identification of therapeutic targets, such as small molecules, that modulate the interaction of DUX4 with DNA [86], but this will be discussed later in this review. However, this binding appears to be mediated by the folding of homeodomains that cover three consecutive grooves and attach to DNA, which is unusual because this has never been observed in other transcription factors with α-helical bond domains [87,88].

### 5.1. DUX4 Mechanisms in Embryogenesis

After fertilization, DUX4 accumulates with a peak at the intermediate stage and then decreases [89,90], a factor which plays an important role in embryonic development. DUX4 is essential in embryology because it directly activates the transcription of genes encoding proteins that are transiently expressed in embryos [89]. Several targeted sequencing studies have shown that DUX4 is able to act by directly activating the transcription of protein-coding genes that are transiently expressed in embryos, in particular: lysine demethylase 4E (*KDM4E*), zinc finger and SCAN domain containing 4 (*ZSCAN4*), preferentially expressed antigen of the melanoma family (*PRAMEF*), and zinc finger protein 352 (*ZFP352*) [89]. Ectopic *DUX4* expression in embryonic cells is sufficient to drive the transcription of zygotic genome activation (*ZGA*) genes [89,90,91,92]. As a result, the expression of *DUX4* is tightly controlled at the DNA, RNA, and protein levels, and its failure to deactivate could result in developmental alterations with the development of pathologies and even embryo death (Figure 3) [93].

### 5.2. DUX4 Mechanisms in Post-Natal Development

After the embryonic stage, *DUX4* is silenced in most somatic tissues, except for the thymus and testis, where RNA and protein expressions are physiologically high [14]. It is unknown which role DUX4 plays in these tissues; however, it has been hypothesized that it is linked to the high level of apoptosis that distinguishes them, so research is still on-going to better understand this aspect [94]. A point in favor of this theory can be found in certain papers, in which a mouse homolog of human DUX4 is expressed and plays a role in the elimination of cells that turn out to have a wrong formation of the T-cell receptor [95]. In order to understand its target genes, many studies on the ectopic expression of *DUX4* have also been performed in somatic cells with gene sequencing [96,97], although the results appear to be uncertain, indicating the need for more in-depth research. Gene ontology analyses of *DUX4*-regulated transcripts have revealed a change and hence involvement in numerous processes, including proliferation and differentiation, RNA processing, the regulation of immune response, viral response, and lastly cytoskeletal arrangement (Figure 3). To summarize, the embryonic mechanism of action of DUX4 and some evidence explored in somatic tissue are incompatible with normal myogenesis due to the stimulation of apoptosis that is triggered by the typical altered expression of DUX4, which results in the typical dystrophy phenotype. Furthermore, we mention the direct relationship between DUX4 and various transcription factors, such as double homeobox A (DUXA), double homeobox B (DUXB), ZSCAN, or leucine twenty homeobox (LEUTX), as well as methyl-CpG binding domain protein 3-like 2, 3, 5 (MBD3L2, MBD3L3, MBD3L5) or KDM4E. These factors can influence each other, also acting in turn on the transcriptional cascade induced by DUX4 [98]. DUX4 targets include the histone variants H3.X and H3.Y described later in this review.

In addition to the genes mentioned above, DUX4 can also bind and stimulate the transcriptional activation of normally silenced repetitive elements, such as mammalian apparent retrotransposons (MaLRs), endogenous retroviruses (ERVs), and human pericentromeric satellite repeats II (HSATIIs) [96,99,100]. Some MaLRs and ERVs that are activated by DUX4 generate novel promoters for genes, long non-coding RNAs, and antisense transcripts. Because they are expressed in FSHD muscle cells but not in the control ones, several of these new transcripts may contribute to the pathophysiology of FSHD [100]. During mammalian evolution, these transposable elements could modify the lineage-specific patterns of gene expression via DUX4 [100].

### 5.3. DUX4 Regulation Mechanisms

**Figure 4:**
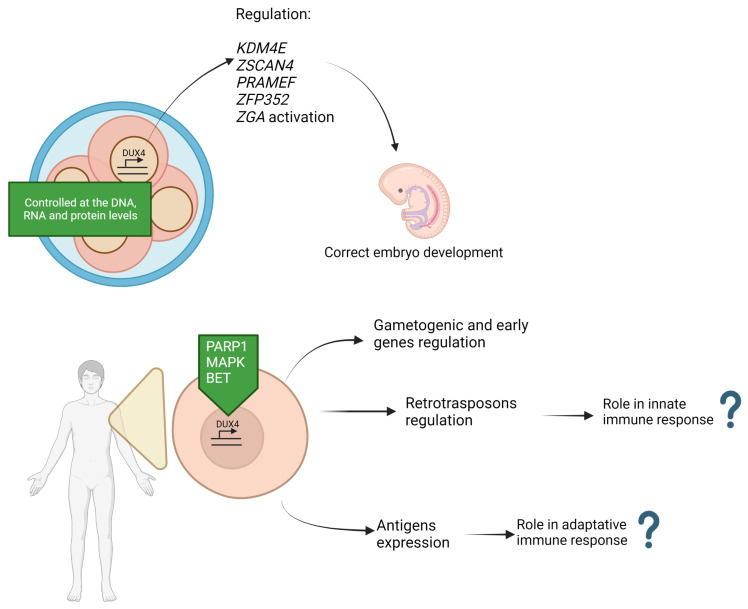
DUX4 activity in activations of mechanisms of embryogenesis, retroelements, and immune protein expression, which are involved in pathogenesis of FSHD.

The transcriptional regulation of DUX4 expression may also be influenced by gene regulatory proteins that interact with the DUX4 promoter, including poly(ADP-ribose) polymerase 1 (PARP1) [101]. *DUX4* target candidates have been silenced in primary FSHD muscle cells using bromodomain and extra-terminal domain (BET) inhibitors (BETi) targeting all the proteins of the BET family (Figure 4) [102]. Their expression was suppressed after treatment with β2 adrenergic receptor agonists, suggesting the role of BET and β2 adrenergic receptor signaling pathways in *DUX4* expression in patients [102,103]. In FSHD muscle cells, inhibiting p38 induced a decrease in DUX4 levels [104]. By lowering the expression of *DUX4* and its target genes, *ZSCAN4* and tripartite motif containing 43 (*TRIM43*)*,* phosphodiesterases (PDEs), which control the amount of available cyclic adenosine monophosphate (cAMP) in the cell, were identified to be DUX4 expression regulators [103].

Pathologies associated with DUX4 have been linked to impaired *DUX4* expression and reactivation in the post-embryonic phase [105,106,107]. As previously stated, the main disorder associated with DUX4 is FSHD. In this kind of dystrophy, DUX4 has been associated with the activation of toxic pathways for muscle [108], including DNA damage [109], the inhibition of myogenic differentiation and oxidative stress [52,110,111], and inflammation [96], favoring myoblast apoptosis [52,112].

### 5.4. DUX4 Involvement in Chromatin Regulation

As previously stated, epigenetic regulation plays an essential role in the disease pathogenesis, which is critical for maintaining cellular homeostasis and muscle differentiation. These processes control the regulation of chromatin structure and condensation. There is evidence in the literature revealing how DUX4 participates in these epigenetic processes and that it has a link with proteins involved in the morphological regulation of chromatin [113]. Maehara and collaborators discovered that in FSHD DUX4 muscle biopsies, histones H3.X and H3.Y are upregulated compared to control muscles. In this study, it was also found that inducing the expression of *DUX4* produces a significant increase in the expression of H3.X and H3.Y [114]. Another intriguing finding is that DUX4 target genes, specifically *TRIM43* and *ZSCAN4*, are not upregulated after H3.X and H3.Y silencing and DUX4 induction. This first study revealed that the presence of H3.X and H3.Y in the loci of the target gene *DUX4* induces the altered and detrimental expression of those genes, favoring the dystrophic phenotype [114]. Other studies had the same results on the link between DUX4 and histone proteins, confirming and clarifying the essential role that DUX4 plays in regulating the proteins involved in chromatin remodeling and that, as a result, the aberrant expression of this protein causes a cascade of epigenetic events that favor the FSHD phenotype [115]. Another recent study demonstrating the importance of DUX4 in proper histone function showed how the iP300w inhibitor (which will be discussed in the following chapters) can prevent the increase in the expression of acetylated histone H3 induced by DUX4 upregulation in dystrophic myoblasts. This study confirmed previously published findings and demonstrated how this inhibitor might prevent epigenetic issues caused by toxicity produced by DUX4 overexpression in the pathogenesis of FSHD [116].

### 5.5. DUX4 Functions in Human Diseases

In recent years, there has been renewed interest in this gene in the field of oncology, as DUX4 regulates an aberrant program of embryonic gene expression that promotes the formation of cancer cells. In particular, it can suppress the MHC class I antigen presentation pathway and immune evasion, thus instructing the immune system to recognize and fight cancer cells; this would also appear to explain why some types of tumors are resistant to drug therapies [117]. Impaired *DUX4* expression has also been discovered in patients infected with all viruses belonging to the Herpesviridae family, which are associated in a variety of human diseases, including mononucleosis, chickenpox, and encephalitis [118].

DUX4c (centromeric or contracted) is a DUX4 homologue that is located 42 kb proximal to the D4Z4 repeat array on chromosome 4, close to the *FRG2* gene. DUX4c is a truncated and inverted D4Z4 unit that shares a major portion of the coding and proximal sequences (including the two homeoboxes) with its homologues. Indeed, the DUX4c ORF is 1125 bp longer than the DUX4 ORF, and both genes share a 1137 bp fragment beginning 111 bp upstream of their shared start codon (MAL), with three mismatches outside the double homeobox domain [119]. DUX4c is actively transcribed and translated, and it has been found to be upregulated in FSHD myoblasts. Its characterization has revealed that the mRNA is polyadenylated, despite the absence of a canonical polyadenylation signal. Furthermore, DUX4c has a heterogeneous 3′end, whereas 5′RACE findings indicate that transcription could be driven by both the variant TATAA box and the multiple GC boxes found in the promoter region [119]. The DUX4c ORF encodes a 47 kDa protein with 347 amino acids. By binding to their promoters, DUX4c has been shown to regulate Myf5 [119] and induce myomiR miR-1, miR-206, miR-133a, and miR-133b [120]. DUX4c is primarily found in the nucleus of proliferating myoblasts, whereas it moves to the cytoplasm during myoblast fusion, where it interacts with multiple proteins, such as desmin, α-actinin, and tubulin (all of which have a double-homeobox domain), [121] suggesting that DUX4c may interfere with muscle differentiation by affecting cytoskeleton assembly and disrupting spliceosome complexes. Recently, it has been found and validated that DUX4 can interact with several RNA-binding proteins (RBPs), such as complement C1q binding protein (C1QBP), serine- and arginine-rich splicing factor 9 (SRSF9; a known C1QBP interactor), RNA-binding motif protein 3 (RBM3), fused in sarcoma/translocated in liposarcoma (FUS/TLS), and splicing factor proline- and glutamine-rich (SFPQ). All of these are involved in the regulation of mRNA splicing and translation [122]. Another study demonstrated that the interaction between DUX4c and RBPs may play a role in muscle cell differentiation, repair, and mass maintenance particularly during the myoblast fusion stage. They also suggested that increased amounts of DUX4c or DUX4 proteins in FSHD muscle cells may obstruct C1QBP or other RBPs from performing their normal functions and impede the process of muscle regeneration, exacerbating the muscle pathology activated by DUX4 [123].

In this review, we will focus on FSHD1 and in particular on its master gene, *DUX4,* which will be discussed in detail below.

## 6. DUX4 and Protein Partners Associated with Muscle Differentiation

DUX4 can activate multiple target genes which in turn seriously impair muscle homeostasis [2,124]. These are involved and alter important pathways, such as immune response and apoptosis [125], leading to oxidative stress [109] and altered myogenesis, which favor the development of severely atrophic muscles [126,127,128]. Several recent studies have also demonstrated the presence of these traits in FSHD muscle [129,130]. The alteration of other myogenesis-related proteins, including MyoD, depends on the low-level expression of *DUX4*. In addition, it also appears to affect other MRFs: myogenin, Myf5, and MRF4 [110,128]. It has been shown in transgenic animal models that the overexpression of *DUX4* strongly alters MyD and myogenin, which is thought to be the molecular trigger for the incorrect differentiation pathway. The same study hypothesized that the expression of Myf5 does not appear to be able to prevent this process [131]. These data regarding the role of transcriptional activity in myoblast differentiation have also been confirmed in humans [97,131,132]. Moreover, Knopp and colleagues found that DUX4 can decrease gene expression for the myogenic response in SCs, thus blocking proliferation and differentiation, as well as myotube fusion [131].

Furthermore, paired box 7 (PAX7) represents a very important factor in the early stages of differentiation and then gradually decreases. This transcription factor is unique in that it shares a DNA binding site with DUX4 in its amino acid sequence [52,128,133,134,135]. This peculiarity may be important for proper myogenesis and suggests that PAX7 plays an important role in FSHD [134,135,136]. These factors were not found to be coexpressed in the muscle of FHSD patients. Previous research has shown that this simultaneous lack of expression promotes myocyte formation from cultured satellite cells of patients with FSHD [134,135,137].

An interesting study published in 2015 demonstrated that *DUX4* expression in FSHD muscle increases with myogenic differentiation in several cell cultures. In particular, the study’s authors examined *DUX4* mRNA expression and discovered an increase during muscle differentiation. Moreover, the same trend was found in the DUX4 target gene *ZSCAN4*. This study also showed that *SMCHD1* is downregulated during myoblast differentiation, emphasizing the role of SMCHD1 in suppressing DUX4 activity in somatic cells and its decline during muscle cell differentiation, as well as promoting *DUX4* expression during myogenesis [138].

Furthermore, the upregulation of DUX4c during differentiation and aberrant intracellular localization of DUX4c were observed in FSHD cells compared to healthy muscle cells: FSHD myotubes still exhibited nuclear and cytoplasmic DUX4c-positive signals [121]. This may then promote the abnormal muscle differentiation typically seen in dystrophy.

Differentiation defects in FSHD muscles have been previously described, such as the inhibition of *MYOD1* target genes [134,139] and genes important in normal myogenesis, such as the encoding proteins associated with muscle structural proteins and stress responses [140]. DUX4 can influence myogenesis at the transcriptional level [52,96] and by interacting with proteins that regulate these genes, such as myogenin (*MYOG)* (as already discussed at the beginning of this section), which is upregulated in FSHD myoblasts compared to healthy immortalized ones [98]. DUX4 does, however, interact with proteins involved in cytoskeleton assembly, such as desmin [141]. Because the cytoskeleton plays critical roles in cell proliferation and signaling, both of which are important in myoblast fusion, defects in it are identified in a variety of muscle diseases [121]. Vanderplanck and collaborators found more evidence of DUX4c involvement in appropriate cytoskeletal organization and nuclear distribution in FSHD myotubes [142]. Their study shows how inhibiting DUX4 or DUX4c can improve abnormalities by alleviating dystrophic muscle conditions. Furthermore, the study demonstrated how the abnormal expression of *DUX4* is clearly involved in the correct process of muscle differentiation [123,142].

However, DNA damage and oxidative stress induced by *DUX4* overexpression also appear to be responsible for the aberrant muscle differentiation observed in FSHD. Dmitriev et al., demonstrated how treatments with antioxidant agents improve human muscle cells, notably in primary cultures, and discovered that these treatments during differentiation result in a significant enhancement of this process [109].

## 7. DUX4 and FSHD: Possible Treatment Strategies

Although it has been known for several years that the alteration of *DUX4* is responsible for FSHD, there are still no treatments and therapies that improve the condition of FSHD patients. For this reason, several clinical studies have been conducted but with no clear results [104,143]. In this chapter, we attempt to highlight the most significant approaches currently known and to analyze them, dividing the possible treatments into molecular therapies and possible pharmacological strategies (Table 2).

### 7.1. Molecular Treatment Strategies

As already mentioned, BETi blocks DUX4 expression by preventing bromodomain-containing protein 4 (BRD4) from binding to acetylated lysines while enabling HDAC1/2 access to the exposed acetyl groups. BRD4 acts by binding to acetylated open chromatin, where it recruits transcription-regulatory complexes to enhance transcription [102].

In recent years, many research groups have been addressing another type of treatment, in particular the use of antisense oligonucleotides (AONs) [121,147,148] that comprise different classes of molecules able to block the transcription and/or translation of *DUX4* mRNA through the RNAse H1 pathway. In a transgenic mouse model, the systemic administration of AONs targeting the *DUX4* transcript resulted in significant reductions in DUX4 protein and mRNA levels as well as DUX4 target gene expression. Mice receiving the DUX4-targeted AON displayed reduced skeletal muscle degeneration and fibrosis, displayed less inflammatory dysregulation, and outperformed control mice on various functional outcome measures, particularly those related to muscle strength, suggesting the use of AONs as a promising therapy approach for FSHD [144,149]. Moreover, it emerged from a study of Lim et al., that *DUX4* pre-mRNA levels can be decreased by inhibiting the nucleic acid gapmer (LNA) [150]. These LNAs are a modified RNA-nucleic-acid-like class that carries an additional methylene bridge, boosting their affinity for complementary RNA sequences [151]. *DUX4* pre-mRNA is reprocessed in the cell via post-transcriptional pathways [15]. In human myoblasts, it was found that this technique significantly reduces the expression of *DUX4*, similarly to in FLExDUX4 mice, a mouse model mimicking FSHD [3,152]. The results of this research have led to advantages in terms of fusion and muscle structure. Furthermore, injections into mice have demonstrated that LNA treatment reduces *DUX4* expression [150]. 

The next potential molecular treatment that we will discuss in this review is the usage of micro(mi)RNAs, although this technique still needs to be improved. MiRNAs play important roles in cellular processes, such as cell differentiation. They are also capable of silencing genes, such as *DUX4*, targeting mRNA, and inhibiting synthesis [153,154,155,156]. This strategy has recently been used in mice in an effort to silence *DUX4*. Although these innovative techniques still need to be refined, a study has shown results to aid RNA interference (RNAi) gene therapy for FSHD [157]. When utilized as a gene therapy in an FSHD animal model, endogenous human miR-675 was found to suppress *DUX4* mRNA in vitro and exhibit muscle-protective effects. Numerous small compounds are able to boost miR-675 endogenous synthesis, including melatonin and estrogen, making them attractive therapeutic targets [145]. However, the use of this technique remains to be further explored, as there are several aspects to consider when evaluating the potential side effects, such as whether it could deteriorate muscle tissue by triggering the immune system or favoring other cytotoxic processes [158].

Modifying *SMCHD1* is another silencing strategy being studied in FSHD. The CRISPR-Cas9 technique was used to repair a *SMCHD1* pathogenic intronic variant, resulting in the inclusion of a pseudo-exon that led to *SMCHD1* haploinsufficiency. Its deletion mediated by genome editing restored wild-type *SMCHD1* mRNA to a near-normal level and reduced the expression of *DUX4* and its target genes [79]. The CRISPR-Cas9 strategy has been recently applied to an adenine base, editing the functional ATTAAA polyadenylation signal (PAS) in exon three of *DUX4*, which is required for the stable synthesis of full-length *DUX4* mRNA in skeletal muscle [146]. The authors found that this genome-editing approach successfully disrupts a PAS motif, resulting in a decrease in *DUX4* mRNA levels and its transcription factor activity [146]. However, delivering genome editing techniques to muscle cells still remains a significant challenge that must be overcome to definitively validate this method. Moreover, the applicability of the CRISPR-Cas9 approach requires locus-specific validation.

A novel inhibitor of p300-mediated H3K9 acetylation, iP300w, was recently tested as well. It has the ability to inhibit the cytotoxicity of the DUX4 protein and reverse the overexpression of DUX4 transcriptional targets in both in vitro and in vivo models of FSHD [116]. Unfortunately, this treatment has been shown to induce toxic changes to control cells without overexpressing *DUX4*. This inhibitor needs to be studied more carefully, because it may have harmful effects on the patient and be associated with unpleasant side effects [116]. Nevertheless, this is an important proof of concept supporting the search for selective inhibitors of DUX4’s interaction with p300.

### 7.2. Pharmacological Treatment Strategies

As stated above, it has been recently discovered that MAPKs, specifically p38, regulate *DUX4* expression [104,159]. In particular, p38α appears to be the main p38 isoform involved in the inflammatory response [160,161] and also functions as a molecular regulator for muscle differentiation. As mentioned in the previous chapter, p38α regulates the markers of myogenesis and DUX4 molecular targets [162]. Many studies have shown that clinically advanced p38 inhibitors are capable of downregulating *DUX4*, thus demonstrating that its expression is sensitive to p38 inhibition [159], although the molecular mechanisms binding DUX4 to p38 remain unclear. Investments are being made in p38 inhibitors to assess the modulation of *DUX4* and, consequently, the alterations in the FSHD muscle as potential pharmaceutical targets. A study carried out by Fulcrum Therapeutics/USA tested the p38 MAPK inhibitor losmapimod in patients with FSHD. Eighty patients were enrolled in a phase-2b randomized, double-blind clinical trial to investigate the effects of oral losmapimod administration. The treatment revealed a clinically relevant advantage over a placebo in terms of different functional and patient-reported outcome measures. This drug was also well tolerated, and no significant side effects were observed [163]. Moreover, the losmapimod treatment resulted in a significant decrease in *DUX4*-driven gene expression, whereas muscle biopsies taken before treatment revealed high levels of *DUX4* expression [164]. These outcomes confirm previous molecular studies on the sensitivity of DUX4 to p38 inhibitors.

As an increasing amount of evidence has indicated that oxidative stress can contribute to the disease pathophysiology, the use of antioxidants is another intriguing treatment that can control or delay oxidative insults and potentially aid in maintaining FSHD muscle function. In vitro studies have already demonstrated the obvious advantages of antioxidant compounds on the morphology and phenotype of dystrophic muscle, increasing its resistance to pro-oxidant-mediated damage [165]. Furthermore, the use of the antioxidant tempol improved myotube formation by decreasing DUX4-induced cytotoxicity [109].

Along with the use of selenium and zinc, the effects of vitamins C and E on dystrophic patients have also been examined. Recent work has shown that these co-treatments enhanced muscle function in patients with FSHD [166]. However, there is still a lack of clinical evidence about antioxidants being used to treat patients with FSHD. Further research will be needed.

Clinical observations are critical for moving clinical trials forward and securing treatment approval. Analyses of FSHD are very important, since they are also designed to assess muscle and physical function in FSHD patients. This is significant because, whereas the tests used for people with other neuromuscular disorders and dystrophies are typically very comparable, they are not always totally appropriate for patients with FSHD [167]. However, specific measurement methods for FSHD have been developed in recent years and many more are in the pipeline. These include functional outcome measures, particularly the FSHD Composite Outcome Measure (FSHD-COM) and Reachable Workspace (RWS) [168,169]. Furthermore, two specific parameters, the FSHD Health Index (FSHD-HI) and FSHD-Rasch Constructed Overall Disability Scale (FSHD-RODS), are still used to evaluate the clinical treatment of FSHD [59]. These aspects, however, will not be discussed in this review but are mentioned to emphasize the significance of the development and study of new therapeutic options, both clinical and molecular, for this complex disease. In addition, the alternative strategies described above and those that have been used to date are based on these clinical outcomes.

### 7.3. Possible Cancer Therapeutic Treatments Applicable for FSHD

As already mentioned in the preceding paragraphs, DUX4 has recently been discovered to have an altered expression also in a variety of cancers. In addition, several investigations are being conducted in this field of study to determine how to act on the possible modulation of DUX4 in tumor cells. In this final brief paragraph, we will examine some recent intriguing results that could be investigated and applied in future studies on the modulation of DUX4 in muscle cells affected by FSHD.

Ponatinib, an inhibitor of several tyrosine kinases, is the first chemical utilized in oncological therapy. There are ongoing studies to determine whether this inhibitor plays a role in the control of *DUX4* expression as well as muscle alterations in FSHD patients. Indeed, this inhibitor acts on several kinases involved in the regulation of DUX4 also in muscle cells [170]. Proteostasis inhibitors, such as carfilzomib and bortezomib, are also commonly utilized in cancer research and are of interest for DUX4 regulation [171,172,173]. Alterations to the protein degradation pathway, particularly changes to the proteasome’s correct functioning, have long been suspected of being involved in the start of the dystrophic phenotype, as these processes create protein clumps that injure muscle cells [174,175]. These are some of the chemical compounds used in oncology that have also been shown to inhibit DUX4 in cancer cells [170,176]. Because they act on cellular pathways that are disrupted in this kind of dystrophy and in DUX4, they may play an important role in future scientific studies to develop additional therapeutic options for this neuromuscular disorder.

## 8. Conclusions and Future Perspectives

In this review, we discuss differentiation and regeneration in muscle tissue, analyzing some altered cellular and molecular aspects typical of FSHD, the third most common form of muscular dystrophy. It is well known that FSHD is caused by a number of distinct genetic and epigenetic abnormalities, all of which result in the aberrant production of the *DUX4* transcription factor. When expressed, DUX4 triggers a series of events that culminate in cell death, which hinders muscle growth and repair [135]. It has recently been discovered that DUX4 can interact with MRF proteins, altering their expression and causing incorrect myogenesis. Cellular and molecular studies are important to better understand the still unknown function of DUX4. All of this is critical for identifying new regulators of *DUX4* expression and attempting to block its aberrant expression, which results in the blockage of the pathways in which it appears to be involved. Furthermore, recent studies, which we have discussed above, may aid in the development of inhibitory therapeutic strategies. We attempt to summarize the most recent major discoveries of new inhibitors that could represent promising future therapeutic opportunities in this field. We have also discussed some gene therapies that are being researched in this field and that are still being continuously improved and updated today. Finally, we have summarized the few pharmaceutical strategies that have received the most attention in FSHD to date. In this regard, a number of researchers are developing treatment strategies that target DUX4 by either preventing the production of *DUX4* mRNA [103,177] or by employing AONs to specifically target *DUX4* mRNA [147,178,179]. The first DUX4-targeted treatments are either already under investigation or are proceeding to in vivo testing and eventual clinical approval. Of them, one phase-2 clinical trial of losmapimod (NCT04003974) which attempts to suppress or reduce *DUX4* expression in skeletal muscle is already ongoing, leading us to better understand the function of DUX4 in the pathogenesis of FSHD [180].

In conclusion, more research is needed to fully understand the DUX4-mediated scenery complex that underpins FSHD and to develop effective treatments capable of correcting the cellular and molecular defects that cause serious damage to muscle homeostasis.

## Figures and Tables

**Table 1:** Myogenic regulatory factors (MRFs) related to FSHD.

MRFs	Functions	Reference
MyoD	- contributing to myoblast determination, which is activated in proliferating myoblasts before overt differentiation; - converting fibroblasts into myoblasts and promoting the formation of multinucleated myotubes	[27,28]
Myf5	- contributing to myoblast determination, which is activated in proliferating myoblasts before overt differentiation	[27,28]
MRF4	- contributing to myoblast differentiation and acting downstream of *Myf5* and *MyoD*; - regulating the homeostasis of myofibers	[27,28]
Myogenin	- contributing to myoblast differentiation and acting downstream of *Myf5* and *MyoD*; - regulating myocyte fusion during development; - regulating the homeostasis of myofibers	[27,28]

**Table 2:** Main treatment strategies targeting DUX4 in FSHD.

Treatment	Therapeutic Strategy	Mechanism of Action	Cell/Tissue Model	Relevant Result	Reference
Molecular	BETi	blocking the binding of BRD4 to acetylated lysines	patient-derived immortalized FSHD myoblasts	↓ *DUX4* and its target gene mRNAs	[102]
	AONs	inhibiting translation of *DUX4* mRNA and promoting its degradation	FLExDUX4 transgenic mouse model	↓ *DUX4* and its target gene mRNAs; ↓ DUX4 protein; ↓ skeletal muscle pathology	[144]
	miR-675	RNAi targeting *DUX4* 3′UTR	HEK293 cells transfected with *DUX4* and mice overexpressing *DUX4* by AAV	↓ *DUX4* and its target gene mRNAs; ↓ DUX4 protein; ↓ DUX4 transactivation in vitro; ↓ skeletal muscle pathology in vivo	[145]
	CRISPR-Cas9	editing of pathogenic intronic *SMCHD1* variant	patient-derived immortalized FSHD myoblasts	↓ *DUX4* and its target gene mRNAs; ↑ wild-type *SMCHD1* mRNA	[79]
		base editing of the *DUX4* PAS motif	patient-derived immortalized FSHD myoblasts	↓ *DUX4* mRNA and ↓ DUX4 transcription factor activity	[146]
	iP300w	inhibiting p300-mediated H3K9 acetylation	myotubes from FSHD myoblast clonal cell lines and iDUX4pA mouse model	↓ DUX4 target gene mRNAs; ↓ global H3 histone acetylation	[116]
Pharmacological	losmapimod	inhibiting p38 MAPK signaling	patient-derived immortalized FSHD myotubes/myoblasts and FSHD xenograft mouse model	↓ *DUX4* and its target gene mRNAs	[104]
	antioxidants	Superoxide radical scavenging (tempol)	primary FSHD myoblasts and myotubes	improving myotube formation of FSHD myoblasts; ↓ DNA damage in FSHD myotubes	[109]

AONs, antisense oligonucleotides; BETi, bromodomain and extra-terminal domain (BET) inhibitors; BRD4, bromodomain-containing protein 4; PAS, polyadenylation signal; RNAi, RNA interference; SMCHD1, structural maintenance of chromosomes flexible hinge domain-containing 1.

## Data Availability

Not applicable.
